# Pediatrics

**Published:** 1899-04

**Authors:** Maud J. Frye

**Affiliations:** Clinical instructor in diseases of children, Medical Department, University of Buffalo


### PEDIATRICS.

Reported by MAUD J. FRYE, M. D.,
Clinical instructor in diseases of children, Medical Department, University of Buffalo.

### TREATMENT OF THE FEVER OF PNEUMONIA IN CHILDREN.

HENRY DWIGHT CHAPIN, in a paper read before the pediatric section of the New York Academy of Medicine, (*Medical News*, November 19, 1898,) says: "Fever *per se* does not call for treatment. The indication for treatment is not the degree of fever, but the disturbance the fever is causing. Phenacetin is the only coal tar preparation to be used; antipyrin and acetanilid never. The application of water is on the whole the safest and most satisfactory method of controlling dangerous hyperpyrexia. Cold to the head will sometimes cause reduction of temperature. Finely cracked ice placed in bladders from which the surplus air has been expelled may be molded around the head, especially at the vertex and occiput, or an ice poultice made by mixing finely cracked ice with linseed meal in oiled silk may be used. If cold to the head does not accomplish the reduction of temperature, compresses may be applied directly to the chest. The child is stripped, wrapped in a blanket and placed on a table. A stimulant is given and the feet are placed in contact with a hot water bottle. A compress sufficiently large to surround the chest, and slit at the axillary portion to permit fitting, is wrung out of water, ranging in temperature from 70° to 95° and applied to the chest every ten or fifteen minutes till the desired result is obtained. With a high temperature, water at a temperature of 95° or even warmer is used at the start. The temperature of the water may be lowered to 70° or even to 60°. It is often advantageous to use one-fourth alcohol. So long as the feet and hands are warm the compresses may be applied. When the temperature is reduced to 102° or 103° do not renew compresses, but leave them on. Apply compresses with as little disturbance as possible, by tucking them in from the front until they meet behind. With cyanosis with prostration and hyperpyrexia use a warm bath (100°F) with friction."

---

### PNEUMONIA IN VERY YOUNG CHILDREN.

L. Emmett Holt, in a paper read before the New York Academy of Medicine, among other things said : "Three types of the disease may be fatal. In the first class the patient may die of exhaustion, because of diminished resistance on account of previously debilitated condition; second, the death may occur from complications; third, from acute toxemia. In the first class of cases the most important points in treatment are an abundance of fresh air, intelligent nursing and careful feeding. Control the cough by inhalations. Stimulation is usually required after a few days. For fever use the ice cap or sponging. Early in the disease use moderate counter-irritation to the chest. Do not give a single dose of medicine, unless demanded.

In cases characterised by profound toxemia, cardiac and respiratory stimulants are required early. These in order of their value are strychnine, nitroglycerine, oxygen, alcohol, caffeine. Strychnine may be given in doses of 1-200 gr., every three hours to a child of one year. Nitroglycerine is especially useful early with pale face, feeble pulse, cold extremities, and signs of pulmonary engorgement. Give 1-400 gr. every hour and after two or three doses at four to five hour intervals. Alcohol though very useful is apt to be pushed too far. It is seldom necessary to give more than one ounce of brandy daily. Caffeine in 1-10 gr. doses is sometimes better than strychnine in dull drowsy patients." Abstracted from an article in the *Medical News*, November 19, 1898.

